# Children’s participation in the development and evaluation of parent-led interventions for children’s mental health: a scoping review and consultation with children

**DOI:** 10.1007/s00787-026-03187-0

**Published:** 2026-09-15

**Authors:** Rebecca Nowland, Georgia Aspinall, Danielle L. Christian, Kathryn Berzins, Deborah J. Crook, Sundus Khalid, Shalini Ahuja, Lucy Gallagher, Brioney Gee, Ella Mickleburgh, Blossom Fernandes, Becca Randell, Afra Kelsall, Daniel Michelson

**Affiliations:** 1 Manchester Institute of Education, University of Manchester, Manchester, UK; 2 NIHR Applied Research Collaboration Northwest Coast, University of Lancashire, Preston, UK; 3 School of Health, Social Work and Sport, University of Lancashire, Preston, UK; 4 Institute of Psychiatry, Psychology and Neuroscience, King’s College London, London, UK; 5 Methodologies Research Division, Faculty of Nursing Midwifery & Palliative Care, King’s College, London, UK; 6 Norfolk and Suffolk NHS Foundation Trust, Norwich, UK; 7 Norwich Medical School, University of East Anglia, Norwich, UK; 8 Department of Health Services Research and Policy, Faculty of Public Health and Policy, London School of Hygiene and Tropical Medicine, London, UK; 9 Research Department of Clinical, Educational and Health Psychology, University College London, WC1E 6BT London, UK; 10 Health Innovation Kent, Surrey Sussex, Crawley, UK; 11 NIHR Applied Research Collaboration: East of England, Cambridge, UK; 12 NIHR Maudsley Biomedical Research Centre, South London and Maudsley NHS Foundation Trust and King’s College London, London, UK

**Keywords:** Mental health, Parent-led, parent-focused, Intervention, Children, Co-design

## Abstract

Parent-led interventions are used to address child mental health, but it is unclear whether, or how, children have been involved in their development and evaluation. This review describes methods used to involve children (5–12 years), summarising outcomes and reflections and provides recommendations for best practice and further research. Searches were conducted in five databases in October 2023 and March 2025. Searches of key organisation websites, Google searching, and requests for articles via social media were also conducted. Eligible studies involved children (5–12 years) in the development and/or evaluation of parent-led interventions aimed at preventing or treating child mental health problems delivered in any setting. Study findings were combined using narrative synthesis and shared with children in participatory focus groups (17 children aged 8–11 years, 70.6% male). Review and focus group findings were combined into practice recommendations. From 9,647 studies retrieved, 16 were included. Most were evaluation studies (*n* = 12) involving children via self-reported outcome measures. Four studies involved children in development, with methodologies including workshops, interviews and feasibility testing. Reporting of outcomes from the co-design process was limited but revealed the importance of creative, art-based parent-mediated activities and rewards for progress. Early involvement in intervention design, use of interactive child-specific co-design workshops, and involvement of children with lived experience are recommended. While involving children may require increased planning and time, benefits include increased acceptance, credibility, parental engagement and feasibility. Future research should include greater detail on codesign methods and effectiveness, to enable more extensive understanding of best practice.

**Supplementary Information:**

The online version contains supplementary material available at https://doi.org/10.1007/s00787-026-03187-0.

## Background

While the involvement of patients and public representatives in research is increasingly widespread, the inclusion of children in the development and evaluation of interventions aimed at addressing their mental health needs is still limited [1]. This is despite policy and legislation stating the importance of listening to children’s voices on all matters that concern them. The United Nations Convention on the Rights of the Child [2] states that ‘every child has the right to express their views and wishes in all matters affecting them and have their views taken seriously’ [2]. Involving children enables them to positively influence the research, supports shifts in researchers’ attitudes and shapes outputs [3]. Research funders such as the National Institute of Health Research (NIHR), for example, encourage research teams to involve children as ‘partners’, acting as advisors or co-researchers [4]. This approach involves co-production, a process where service users work as partners with the research team to share their expertise, skills, and experience, which in turn influences the design and implementation of the research [5].

Including children in the development and evaluation of health interventions and related research can enhance relevance and improve quality [6–8]. Their distinctive perspectives are shaped not only by age, developmental stage, and peer influences, but also by family and cultural contexts, socioeconomic circumstances, gender and identity, and the environments (both physical and digital) in which they live and interact. These insights can increase the validity and impact of research findings [7], and also illuminate priorities that intervention developers may not have previously recognised as important. Co-developing interventions with children can also strengthen engagement [9] and help to address barriers that limit their access to services. Moreover, incorporating a diverse range of children’s perspectives can support the creation of more inclusive and appropriately tailored interventions [10].

Despite these benefits, the involvement of children in mental health research and intervention design remains a relatively under-developed area [11]. Mental health interventions are still often developed *for* children rather than *with* them, partly due to ethical and practical challenges in engaging minors in research, limited researcher training in participatory methods, and institutional or funding constraints that prioritise adult-led perspectives [12, 13]. Additionally, concerns about children’s capacity to articulate experiences of mental health, or assumptions about their vulnerability and need for protection, can further discourage genuine collaboration, a view that is contested as children are competent and knowledgeable commentators on their own lives and experiences [13, 14].

Children’s involvement in the development and evaluation of parent-led mental health interventions is particularly limited [15–17]. These mental health interventions are characterised by parents receiving training from a professional targeted at improving their child’s mental health, which the parent then implements independently with their child [18]. Conversely, in many child-focused interventions, parents remain the primary source of feedback and outcome evaluation. Addressing this imbalance is important because parents’ reports may not adequately capture children’s internal experiences [19], and interventions developed without children’s input may overlook their priorities or preferred forms of support [20]. Excluding children from contributing to research that directly affects them also runs counter to participatory and rights-based principles [21], and constrains understanding of the mechanisms through which parent-mediated interventions exert their effects.

Further the development of parent-led mental health interventions rarely involves co-production with children. However, emerging studies have used co-design to facilitate children’s involvement in the development of these interventions. Co-design in mental health interventions involves active collaboration of individuals with lived experience to create solutions and services. This approach fosters diverse representation and legitimizes the control over decision-making, which is crucial for shifting power dynamics and ensuring that interventions genuinely reflect the needs, priorities, and cultural contexts of the communities they aim to support. By embedding lived expertise throughout the design process, co-design enhances relevance, promotes trust and increases the likelihood that interventions will be acceptable, engaging, and effective in real world settings [22]. Despite this approach becoming increasingly more common place in health services and academic research still little is known about appropriate methodologies and best practices.

Currently, no reviews have examined children’s involvement in the development and evaluation of parent-led interventions. Emerging studies have involved children in the development and/or evaluation of parent-led interventions for children’s mental health [10, 23–25]. However, there has been no systematic scoping of how children have been involved, or what can be learned from their involvement. This is a significant knowledge gap given the increasing use and development of these interventions [26], for example in the UK, where they are recommended as first-line approaches in clinical guidelines for child conduct problems [27]. Understanding children’s views on parent-led mental health interventions and approaches to their involvement in development and evaluation could inform recommendations for more effective approaches and guide future research.

## The current study

We addressed the current knowledge gap by undertaking a scoping review to collate existing research on children’s involvement in development and evaluation of parent-led mental health interventions, to establish what is already known about their contribution. Scoping review methodology was deemed appropriate because our research question was broad and exploratory and intended to capture available literature on the topic exploring the extent and range of children’s involvement, synthesizing current knowledge and highlighting gaps for future research [28, 29].

Specifically, this review aimed to describe whether, how and to what extent children (aged 5–12 years) have been involved in the development and evaluation of parent-led mental health interventions for common child mental health conditions (i.e., anxiety, depression, ADHD, and disruptive behaviour difficulties). We focused specifically on what methods have been used to involve children, the views of children and/or adults on the methods used, and any known best practice for involving children in parent-focused interventions. We also shared the review findings with children to gather their views on how they believe children should be involved in the design and evaluation of future interventions. We have synthesised the review and consultation findings to make recommendations for future involvement activities and research directions.

## Methods

This review was conducted according to the JBI Methodology for Scoping Reviews [30] and Preferred Reporting Items for Systematic Reviews and Meta-Analyses extension for Scoping Reviews (PRISMA-ScR; reporting checklist is provided in Online Resource 1) [31].

### Protocol and registration

The scoping review protocol was registered with Open Science Framework: https://osf.io/x9376/ and published in *JBI Evidence Synthesis* [32].

### Information sources and searches

The search strategy detailed in Table 1 was developed from the text words used in the titles and abstracts of relevant papers identified from preliminary searches on the topic, conducted in MEDLINE (via OVID), Cumulative Index to Nursing and Allied Health Literature (CINAHL via ESBCO information Services) and Google. Initial searches were conducted in five databases in October 2023 and then repeated in March 2025 including APA PsycINFO (via EBSCO), MEDLINE (via OVID), CINAHL (via EBSCO), Excepta Medica DataBASE (EMBASE, via OVID), and SCOPUS. Searches were adapted to meet the Boolean and truncation operations for each database. Full detailed search strategy is provided in Online Resource 2. Searches were limited to English Language, and no date restriction was applied. Hand searching was undertaken to identify any relevant grey literature and missed articles including Google searching of key terms, searches of key organisation websites (full details are provided in Online Resource 3) and examination of reference lists of included articles. In addition, a request for relevant articles for inclusion was made via social media advertised by authors and through relevant networks.

**Table 1:** Search strategy

Participants	((child*) OR (kid*) OR (girl*) OR (boy*))
Concept	((Experience) OR (Opinion*) OR (View*) OR (Attitude*) OR (Perception*) OR (Perspective*) OR (Involvement) OR (PPI) OR (Child report*) OR (Participation) OR (Child-reporting) OR (Child reporting) OR (Participatory research) OR (Participatory method*) OR (Participatory approach*) OR (Participatory design) OR (Participatory model*) OR (Co-produc*) OR (Coproduc*) OR (Co-research) OR (Coresearch) OR (Co-creat*) OR (Cocreat*) OR (Co-design*) OR (Codesign*) OR (Co-develop*) OR (Codevelop*) OR (Co-investigator*) OR (Coinvestigator*) OR (Advisory group*) OR (Advisory council) OR (Child-led) OR (Child led) OR (Research partner) OR (Consultation))
Context	AND ((Parent-led) OR (Parent led) OR (Parent directed) OR (Parent delivered) OR (Parent-delivered) OR (Parent focus*) OR (Parent-focus*) OR (Parent* adj2 (program*)) OR (Parent* adj2 (intervention*)) OR (parent* adj2 (class*)) OR (parent* adj2 (group*)) OR (parent* adj2 (course))) AND ((Mental health) OR (Anxiety) OR (Depress*) OR (Trauma) OR (Cognitive behavioural therapy) OR (Cognitive behavioral therapy) OR (Cognitive behaviour therapy) OR (Cognitive behavior therapy) OR (CBT) OR (Behavioural problems) OR (Behavioral problems) OR (motivational interviewing) OR (cognitive analytic therapy))

### Eligibility criteria

Inclusion and exclusion criteria are displayed in Table 2. Only peer-reviewed journal articles and grey literature reports were included, using any design where children were involved in the development or evaluation of parent-led interventions. While we narrowed our searching to peer-reviewed journals, as the focus for this review related to the involvement of children in development and evaluations rather than effectiveness of the interventions, we included grey literature reports to capture this involvement in any local practices in commissioned reports.

**Table 2:** Inclusion and exclusion criteria

	Inclusion	Exclusion
Participants	Children from any country, regardless of disability, aged 5–12 years	Studies exclusively with children not aged 5–12
Concept	Studies involving children in the development and/or evaluation of parent-led interventions where the primary object is treating or preventing child mental health problems, specifically anxiety, depression, ADHD, or disruptive behaviour difficulties. Eligible interventions include at least one psychosocial practice element aimed at behavioural, cognitive, emotional, interpersonal, social, and/or environmental factors associated with common child mental health problems.	Parenting and/or family intervention studies and studies where the intervention includes parallel sessions with parents and children.
Context	Any parent-led interventions aimed to support children, provided to parents in any setting (e.g., school, online, health care service).	
Study type	Peer-reviewed journal articles and grey literature reports	Conference abstracts, opinion papers, dissertations and editorials.
Dates	Any publication year	
Language	English	Not English

Any form of children’s participation involving the design or development of interventions (e.g., codesign studies) and in the evaluation of interventions (e.g., feasibility studies, qualitative studies) including providing self-report measures were eligible. In terms of evaluations, studies that involved children as (assenting) participants and gathered their views were included, as well as studies where children were co-researchers or advisors. Eligible studies could have involved children at any stage in the research process.

Eligible parent-led interventions included at least one practice element addressing one or more behavioural, cognitive, emotional, interpersonal, social and/or environmental factors implicated in common child mental health problems [33]. For the study to be included, the primary objectives of the intervention had to be to prevent or treat one or more common mental health problems, specifically anxiety, depression, ADHD, or disruptive behaviour. Studies of interventions involving parallel sessions with children and parents, as well as family intervention studies, were excluded. Parent-led interventions could be delivered to parents in any setting (e.g. healthcare service, school) with training and techniques utilised to support their children in their everyday life (e.g. in a home setting). “Parent” was defined to include any parental caregiver (including biological and non-biological caregivers, e.g. foster parent, social care workers, and kinship care). Studies involving children from any country, regardless of disability, aged 5–12 years (i.e., those in primary or elementary school) because as parent-led interventions are typically aimed at younger children’s mental health [34]. Given the scarcity of studies that have involved children in the development and/or evaluation of parent-led interventions studies were included that met the criteria but also included children outside of the age range.

### Study selection

All identified papers from database searches were uploaded into Endnote and duplicates removed. Citations were then imported to Rayyan [35] for further duplicate removal and title and abstract screening. Following a pilot test, titles and abstracts were assessed against the inclusion/exclusion criteria. To improve consistency and precision at least 20% of articles were screened independently by two reviewers at the title and abstract screening stage [36]. Inter-rater reliability was examined using percentage agreement and was 95.8% at this stage.

Full texts of potential papers for inclusion were retrieved and full-text screening was conducted in Excel. Full texts were assessed against the inclusion/exclusion criteria, with reasons for exclusion recorded. Where there was data missing in publications that was critical for our review we contacted authors for further details. This involved contacting one author about the ages of children involved in an expert reference group. For added rigour, each of the retrieved 71 full-text articles was screened independently by at least two screeners. Any disagreements that occurred during screening were solved through discussion and involvement of the review team where needed. Inter-rater reliability was lower at this stage (73.1%), often due to a lack of detail and description about the intervention and/or children’s involvement. To overcome this, further information about the intervention was sourced by emailing authors and sourcing further documents online that describe the intervention in more detail to support decision making in the review team.

### Data extraction and synthesis

Data was extracted from included articles using a tool developed by the research team which included study demographics (e.g. specific population, country, design), details of the intervention (e.g., number of training sessions, delivery, content of training sessions), methods of involvement of children (e.g., self-reporting outcome measures, interviews, focus groups) and which research stages they were involved in and the findings related to their involvement (e.g. children’s reflections on their experience, parent or authors reflections of children’s involvement and impacts).

Data were extracted from all included studies independently by two authors who then compared their results to reach consensus through discussion, as needed. Extracted data were organised into a chart [37] providing an overview of the findings, including ways in which children were involved, stages of involvement and adults reflections on that involvement. Charted findings were then combined into a narrative synthesis [38, 39] to show how, and with what results, children were involved, and identify any themes relating reflections on the involvement.

While critical appraisal is not mandatory in scoping review methodology [40], conducting assessment of methodological quality of included studies was considered but as typical appraisal tools focus on the quality of the research and this review focuses on children’s involvement in the research process. To appraise the studies they were categorized based levels of involvement (e.g. stages that children were involved in the research process) and how they were involved (in development or evaluation).

### Child consultations

We conducted four participatory focus groups in a primary school in a disadvantaged area of Northwest England. These consultations with children aimed to add additional data for the study relating to children’s reflections and suggestions for involving children in the development and evaluation of parent-led interventions for child mental health. A total of 17 children (M: 12; F: 5) participated in the focus groups of which three were 8-year-olds, six were 9-year-olds, five were 10-year-olds, and three were 11-year-olds. To ensure that children who took part in the focus groups had lived experiences of mental health those receiving support with their social and emotional wellbeing within the review inclusion age range were identified to take part by the school Learning Mentor/Safeguarding Officer and parental consent was gained prior to participation. All children were provided with a verbal explanation of a written Participant Information Sheet and asked if they were happy to take part at the beginning of each of the four sessions. Ethical approval was gained from the University of Lancashire for the child consultations (Ethical Approval No. HEALTH 01277).

A schedule for the focus groups was planned ahead of the sessions (full details of the focus group schedule is available in Online Resource 4) but a flexible and adaptable approach was used to meet children’s developmental and individual needs [41, 42]. The focus groups were facilitated by DC and RN who have expertise in child participatory approaches. All focus groups were held on school premises during school time. Each focus group lasted approximately one hour and were not audio-recorded, although notes were made by researchers.

Discussion was supported by sharing two examples of activities (body mapping - noting where emotions are felt in the body and thoughts-actions-feelings triangle) selected as used in some parent-led interventions to help children to conceptualise the interventions. Discussion centred on children’s thoughts about their parents being supported to help them with their mental health, and their views on how children should be asked about their thoughts and feelings about these interventions. Examples of children’s involvement from the reviewed studies were also shared. Children had access to art materials, paper, stickers, and modelling clay in a small and colourful room in the school that they were familiar with and comfortable to be in, usually used for one-to-one or small group discussion. They were encouraged to discuss, draw, model and write down their thoughts and opinions.

In accordance with NIHR Involve guidelines discussion was held with the school about reimbursement to recognise the involvement of the children. It was agreed that vouchers would be provided to the school and that they would discuss a suitable use with the children.

Data from the focus groups with children was analysed using narrative synthesis and combined with the review findings into recommendations for involving children in development and evaluation of parent-led interventions for child mental health.

## Results

**Fig. 1:**
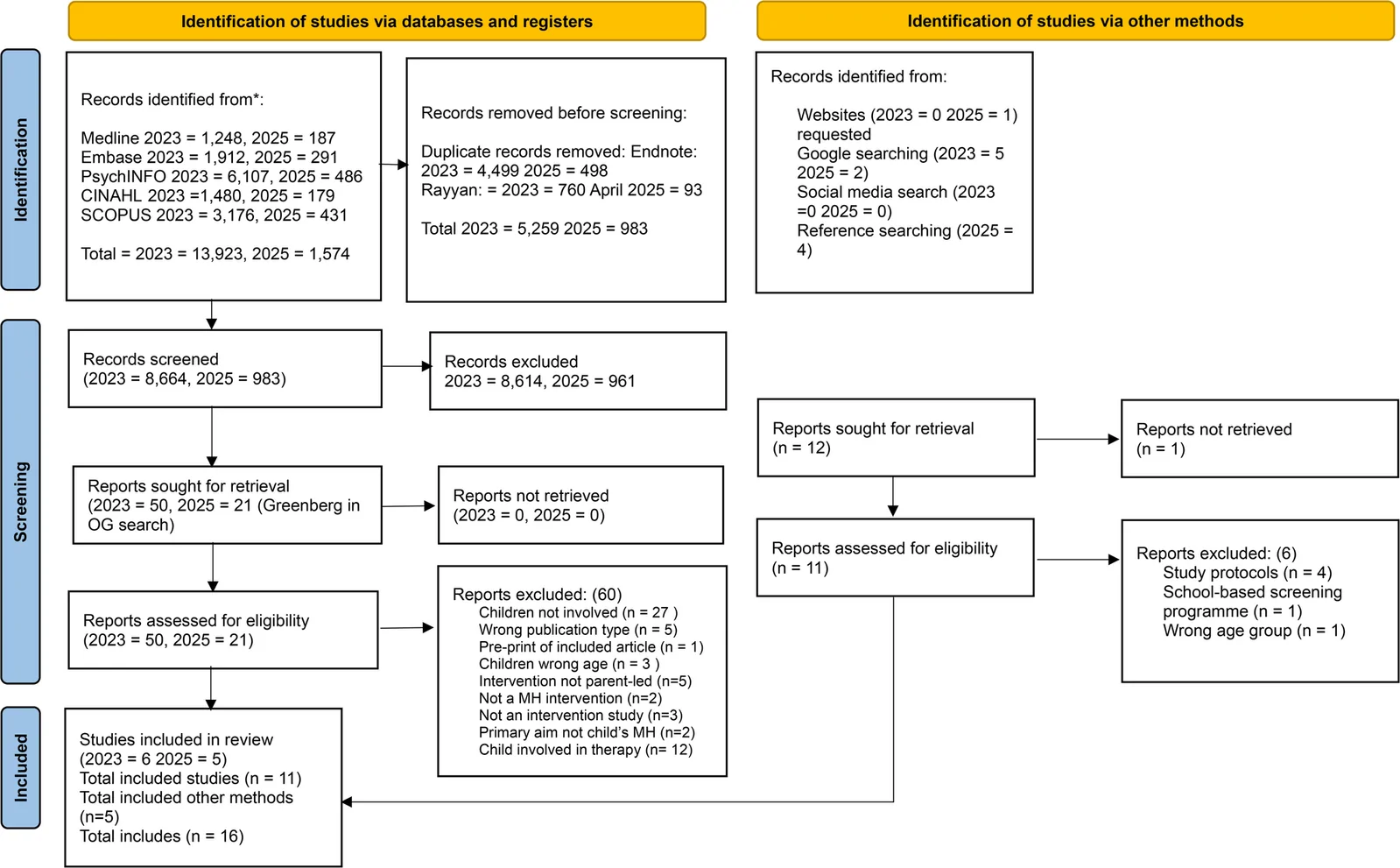
Prisma Flowchart

The results of searches for each year conducted are displayed in a PRISMA flow diagram in Fig. 1. After removing duplicates, 9,647 records were eligible for screening from database searches. Following title and abstract screening, 71 articles were retrieved for full review and assessed for eligibility. A total of 11 studies were considered eligible for inclusion. A further 11 articles were identified through handsearching (i.e. examination of reference lists of included papers, Google searches) of which 5 were deemed eligible when screened, resulting in 16 included studies. All included studies were published in peer-reviewed journals, no relevant grey literature reports were identified.

Most of the studies (*n* = 12) involved children in evaluation of interventions, with four involving children in the design and development of interventions. Descriptive information about the included studies is displayed in Table 3 and raw data extracted for the narrative synthesis provided in Online Resource 5.

**Table 3:** Descriptive information about included studies by study type

First Author, Year	Country	Child Age mean (SD) in years	Child Age range in years	No. of children	% female	Ethnicity (% white)	Universal provision/targeted cohort	Aim of study	Study type	Intervention	Mental health focus	Strategies used to involve children	Research/evaluation stages involved
*Development Studies*													
Armitt, 2025 [10]	UK	NR	5–11 (all < 7)	Coproduction: 9 User testing: 11	NR	NR	Clinical diagnosis of ADHD as reported by parents/ guardians	Co-produce a parent-led nature-based intervention to support mental health and wellbeing and symptom reduction in children with ADHD	Codesign Feasibility	Co-production of a nature-based intervention for children with ADHD study (CONIFAS)	ADHD	Co-production workshops Usability testing Redefining intervention	Intervention design Feasibility testing Refinement of intervention
Cullingham, 2025 [25]	UK	10 (Median)	8–12	9	NR	NR	Autistic children with anxiety	Co-design adaptation of a parent-led intervention for non-autistic child anxiety to meet the needs of autistic children	Codesign	OSI	Anxiety	Co-design interviews	Intervention design
Hill, 2022 [24]	UK	Phase1: 10.25 (1.26) Phase 2: 9.50 (0.58)	9–12	4	50	NR	Children with anxiety	Co-design an internet-based treatment for child anxiety disorders (phase 1) and testing the usability of this internet-based treatment (phase 2).	Codesign	OSI	Anxiety	Codesign workshops Usability questionnaire Interviews	Intervention design Feasibility testing
Pile, 2021 [23]	UK	Co-design focus groups: NR Case series: 8.29 (1.19) Feasibility testing: 7.73 (1.22) Control: 7.79 (1.21)	Co-design focus groups: 9–10 Case series: 6.33–9.73 Feasibility testing: NR Control: NR	Co-design focus groups: 30 Case series: 12 Feasibility testing: 29 Control: 27	Co-design focus groups: NR Case series: 33.3 Feasibility testing: 58.6 Control: 66.7	58	Universal provision	Develop and evaluate a parent-led intervention to target over general memory and avoidance of negative memories	Codesign Pilot Feasibility	My Memory Forest	Emotional disorders	Co-design focus groups Self-report measures Acceptability questionnaire	Intervention design Feasibility testing Participant
*Evaluation studies*													
Breinholst, 2021 [43]	Denmark	8.77 (1.4)	6–12	115	55.6	NR	Universal provision	Test the effectiveness of the treatment program applied in the pilot study in a Danish community	Effectiveness trial	Cool Kids	Anxiety Depression	Self-report measures	Participant only
Chessell 2024 [44]	UK	10.9 (1.1)	9–12	10	70	80	Children who met DSM-5 criteria for OCD	Evaluate the acceptability and efficacy of a brief therapist-guided, parent-led CBT intervention for children with OCD	Efficacy trial	Therapist- guided, parent-led CBT adapted for OCD	OCD	Self-report measures	Participant only
Creswell, 2017 [45]	UK	(Months) GPD-CBT: 111·31 (21·93) SFBT: 109·74 (26·76)	5–12	136	GPD-CBT: 53 SFBT: 53	GDP-CBT: 91 SFBT: 96	Children with anxiety associated with clinical impairment	Compare clinical outcomes and cost-effectiveness of two brief psychological interventions for children with anxiety referred to child mental health settings	Efficacy trial Cost-effectiveness	Brief GPD-CBT & Solution-focused brief therapy	Anxiety	Self-report measures	Participant only
Creswell, 2024 [46]	UK	9·20 (1.79)	5–12	OSI: 222 Treatment as usual: 222	58	OSI: 87 Treatment as usual: 93	Anxiety as a problem determined by clinical teams	Determine whether therapist-supported, digitally augmented, parent-led CBT brings cost savings in routine children’s mental health services without compromising clinical outcomes	Efficacy trial Cost-effectiveness	OSI	Anxiety	Self-report measures	Participant only
Guzick, 2024 [47]	US	10.2 (1.8)	7–13	Low- contact CBT: 48 Standard-contact CBT: 39	18	64	Autistic youth with anxiety	Compare clinical and cost outcomes from two parent led, therapist-assisted CBT telehealth models for anxiety in autistic youth	Efficacy trial Cost-effectiveness	Resource name: Helping Your Anxious Child	Anxiety	Self-report measures	Participant only
Lyneham 2006 [48]	Australia	9.42 (1.96)	6–12	100	49	NR	Children with anxiety from rural/remote communities	Examine efficacy of bibliotherapy in a rural community and the impact of therapist contact using either the telephone, emails, or client-initiated contact.	Effectiveness trial	Resource name: Helping Your Anxious Child: A Step-by-Step Guide for Parent’s	Anxiety	Self-report measures	Participant only
Simon, 2012 [49]	Netherlands	CI: 9.94 (1.28) PI: 9.59 (1.17) NI: 9.96 (1.11)	8–12	CI: 47 PI: 49 NI: 43	CI: 57 PI: 55 NI: 51	NR	Children who scored in as high-anxious on the Screen for Child Anxiety Related Emotional Disorders-71 (SCARED-71).”	Perform an economic evaluation comparing relative costs and outcomes of CI, PI, and NI.	Cost-effectiveness trial	Not reported	Anxiety	Self-report measures	Participant only
Storch, 2024 [50]	US	10.2 (2.7)	7–17	68	48	62	Children and adolescents with clinically significant anxiety and/or obsessive-compulsive disorder (OCD)	Efficacy of the parent-led intervention Supportive Parenting for Anxious Childhood Emotions (SPACE) relative to a low-dose version of the protocol among children and adolescents with clinically significant anxiety and/or OCD	Efficacy trial	Parent-led intervention SPACE	Anxiety OCD	Self-report measures	Participant only
Thienemann, 2006 [51]	UK	12.1 (2.4)	7–16	24	41.7	75	Children experiencing anxiety	Effectiveness of an existing parent-only group CBT protocol to treat children with primary anxiety disorder diagnoses	Effectiveness trial	Cool Kids & Helping your anxious child	Anxiety	Self-report measures Satisfaction questionnaire	Participant Evaluation
Thirlwall, 2013 [52]	UK	NR	7–12	Full: 64 Brief: 61 Waitlist: 69	46.9	Full: 86 Brief: 87 Waitlist: 84	Children with anxiety	Efficacy of low-intensity GPD-CBT treatments for children with anxiety disorders (full, brief and waitlist control trial)	Efficacy trial	GPD-CBT for anxiety	Anxiety	Self-report measures	Participant only
Thirlwall, 2017 [53]	UK	(Months) 120 (19.35)	7–12	125	48	NR	Children with anxiety	Examine predictors of response to GPD-CBT	Secondary analysis	GPD-CBT for anxiety	Anxiety	Self-report measures	Participant only
Van der Sluijs, 2012 [54]	Netherlands	5.58 (0.95)	4–7	26	54	NR	Children with anxiety	Examine the effectiveness of a CBT-based treatment for anxious children	Effectiveness trial	Confident Kids	Anxiety	Self-report measures	Participant only

SD = Standard Deviation; UK = United Kingdom; US = United States of America; NR = Not reported; GPD-CBT = Guided parent delivered CBT; SFBT = Solution-focused brief therapy; OSI = Online Support and Intervention (OSI) for child anxiety; CI = Child-focused intervention; PI = Parent-focused intervention; NI = No intervention

### Characteristics of included studies

Included studies were conducted in the UK (*n* = 10), Netherlands (*n* = 2), United States of America (US) (*n* = 2), Australia (*n* = 1), and Denmark (*n* = 1). Children’s age ranged from 5 [43] to 17 years old [44], with most studies including children aged 8–11 years old. Where gender of children was reported (*n* = 14, 82.4%), the gender spilt was roughly equal except for two studies where 18% [45] and 70% [46] were female. Around half of the studies (*n* = 8, 47.05%) provided information about ethnicity [23, 44–47, 50–52]. All these studies were in the UK or US, and most children were White British/American (range 62–96%).

### Interventions

Of the included interventions only two (11.8%) were designed to be delivered universally for prevention in community samples. One study involved the co-design and feasibility of an intervention to reduce emotional disorders (My Memory Forest) recruiting parents from local primary schools and via an advert on the University’s webpage [23]. This intervention was designed to reduce emotional disorders by targeting overgeneral memory (OGM) using an adaptation of memory specificity training (e.g [51]). and avoidance of negative images. The other study was an evaluation of a therapist-guided parent-delivered self-help group intervention based on the “Cool Kids” program to reduce depression and anxiety recruiting parents from local primary schools via a lecture delivered by authors [52]. All other interventions were targeted at specific populations with mental health difficulties (Anxiety = 11, OCD = 1, Anxiety and/or OCD = 1, ADHD symptom reduction = 1, Emotional disorders = 1).

Six of the 11 studies involving interventions to target children’s anxiety used the Guided parent-delivered CBT for anxiety intervention which uses the *Overcoming your Child’s Fears and Worries: A Self-help Guide using Cognitive-Behavioural Techniques* manual [53] (*n* = 3) [47, 50, 54] or the online version - *Online Support and Intervention (OSI)* for broadly-defined child anxiety (*n* = 3) [24, 25, 47], one of which was focussed on autistic children with anxiety [25]. A further two studies used the text *Helping Your Anxious Child: A Step-by-Step Guide for Parents* [55] to support the intervention [45, 56], one of which was focussed on autistic children with anxiety [45]. Another study took influence from both *Helping your anxious child* and *Coolkids* [49]. The final two studies used the *Confident Kids* intervention [43] and an un-named intervention targeting child anxiety [57].

One study targeted anxiety and/or OCD and used the *Supportive Parenting for Anxious Childhood Emotions (SPACE)* [44] using the SPACE treatment manual [58]. A further study also used the *Guided parent-delivered CBT* in an adapted disorder-specific version intended to treat OCD [46].

Another intervention was targeted at children with ADHD involving a nature-based intervention *Co-production of a nature-based intervention for children with ADHD study (CONIFAS)* [10].

### Involvement of children and outcomes

Only 4 of the included studies involved children in the development of interventions, with the remaining 12 studies involving children in evaluation.

### Development of interventions

Of the four studies which involved children in the development of an intervention [10, 23–25], two also involved children in feasibility trials [10, 23]. Methods used for co-designing the intervention varied including interviews (*n* = 2), focus groups (*n* = 1), workshops (*n* = 2) and usability testing and/or questionnaires (*n* = 4). Studies involving the development of an intervention tended to include older children (see Table 2), with only one study involving participation of children under 8 years old [10].

Cullingham et al. study [25] discussed adaptations of the Guided Parent-Delivered CBT for anxiety for children with autism (OSI). Researchers conducted semi-structured interviews with nine autistic children with anxiety problems and used Interpretive Phenomenological Analysis and Template Analysis to draw out key themes in relation to their experiences and understanding of anxiety problems among autistic children. These findings were used alongside a review of academic literature in an Expert Reference group (ERG; involving one autistic young person aged 16 years with experience of anxiety problems) to create key insights documents. Using the key insights documents, a basic outline of the intervention and case examples were written and then critically reviewed with the ERG. Once a clear intervention outline was established, this was shared with focus groups consisting of parents of autistic children with anxiety to gain further opinions and insights into the acceptability of the intervention. These findings were shared with the ERG and research team and a final outline of the adapted intervention was established.

The study by Hill et al. [24] focussed specifically on the development of a website and app for the online version of the intervention and adopted a user-centred design approach. Children participated in three co-design workshops and three usability testing sessions to give feedback on the overall concept, their wants and needs for the game, and on initial mock-ups of the game. The app was developed in an iterative way based on feedback from each session. During the workshops children were shown game visuals (character type, environment, and style), existing mobile game app types (story-led games, minigames, and virtual toys), and tested out aspects of the game. Children provided their feedback through discussion and by completing a shortened and adapted Program Content and Usability Questionnaire (PCUQ) and were able to vote on which versions/aspects they preferred. The MoSCoW approach (Must have, Should have, Could have, Won’t have) [59] was used to gain insight into the priority of functions preferred by children. In the usability testing sessions, children were presented with a working prototype of the game and asked for their feedback using a usability questionnaire and in semi-structured interviews. Children were asked what they liked and disliked, what was easy to use and what was hard and what they would change. In all workshops children were asked to explore the game and say out loud what they thought of it as they played it (i.e., the think aloud method [60] and observational notes were taken by a researcher.

The study by Pile et al. [23] involved the co-production of parent-led nature-based intervention to support mental health and well-being and symptom reduction in children with ADHD including a co-design study and feasibility trial. This used the Double Diamond process model (Discover, Define, Develop, Deliver [61]) involving a series of workshops with primary school-aged children (*n* = 9), parents/guardians (*n* = 10), and professionals (*n* = 10) were recruited to form the co-production group during the discover, define and deliver phases. In the ‘Discover’ phase, children were asked to describe their favourite and least favourite activities in nature to generate a list of activities that could be included in the intervention. Barriers to engaging with nature (e.g., bad weather) and ways to overcome these barriers were also explored. Some activities, such as sensory exploration of the woodlands and constructing nature treasure boxes, were tested with the children and their parents during the workshops. The research team collected feedback from children in groups and individually recording any notes regarding why activities were liked/disliked, any adaptations that could be made, and collected consensus on which activities should or should not be included in the intervention. The second phase (Define) families provided feedback on how to improve accessibility and usability ready for the user-testing phase (Develop), where a further 11 child-parent dyads trialled the intervention at home over six weeks. These families provided qualitative feedback about their experiences collected via phone calls, a free text questionnaire and diary responses. Finally, in the Deliver phase, user testing feedback was shared with the co-production group, who recommended key changes to the intervention before finalisation.

In the My Memory Forest co-design study, initially 30 children (two groups of 15) were involved in focus groups in primary schools [23]. Each focus group involved (a) discussion around what makes them anxious and sad; (b) understanding of memories and imagery and emotions; and (c) a drawing task to create ideas of characters representing different cognitive processes. Findings from the focus groups were used to help design the intervention, including the format of the storybook and the images and metaphors used. Twelve parent and child dyads then completed the intervention to evaluate its feasibility and acceptability. Following feedback from parents and children, changes were made to the intervention and further co-development was conducted. This involved 15 parent-child dyads who attended a workshop at a local library to confirm the acceptability of changes made to the storybook. After adaptation of the intervention, a feasibility randomised control trial was conducted which also included parent and child self-reporting to evaluate the intervention.

There was relatively little discussion in the included development studies about the outcomes from co-design activities and/or how these were used in the development of interventions. Where outcomes were reported children placed importance on parent-mediated and computer-based tasks within the interventions having creative and game-like tasks such as taking pictures and clay modelling [10]. Children suggested that manuals and books used in the intervention should have pictures and visuals [23]. Adaptability and personalisation were also deemed important factors and children expressed a preference for achieving rewards for progress or for facing their fears in real life [24, 25].

### Evaluation studies

The remaining 12 (75%) studies only involved children in providing self-report outcome measures as part of effectiveness, cost-effectiveness or efficacy trials. Two of these studies involved the same data set [50, 54] from a Randomised Controlled Trial, one reported changes in outcome data [50] and the other examined predictors at baseline of changes in the outcome data [54]. As children’s involvement was confined to completing outcome assessments using child self-report measures, there is no data regarding children’s perspectives on the interventions.

In some evaluation studies, diagnostic interviews were used to support child reporting. In one study, interviewing was considered distressing for children, especially for those who were experiencing severe mental health difficulties, who were happier to participate in interviews with parents [49].

In two of evaluation studies [46, 49] it was reported that children’s self-reporting differed to parent and teacher reporting, highlighting the importance of incorporating child report measures and opportunities to hear children’s opinions directly when evaluating the effectiveness of parent-led interventions. Only one of these studies [49] reported details about the difference between parent and child reporting, noting that children tended to report themselves as non-anxious pre-treatment and parents tended to be overly positive about the effects of the treatment. Van der Sluis et al. [43] point out that credibility and value is added to findings by including child self-report as part of the evaluation process, because their evaluations are less likely to be due to social desirability bias or personal time investments due to not being directly engaged in the delivery of parent-led interventions [43]. They argue that, evidence in their study suggests children are capable of evaluating their own anxiety and detecting change over time [43].

### Reflections on involving children in development of interventions

Methods to involve children in development of interventions varied across the included studies. In the two development studies that used child interviews [24, 25] children were interviewed with their parents present. In some studies authors stressed the importance of holding co-design workshops with children separately from other stakeholders to enable equitable and inclusive participation [10, 24].

Professionals from clinical psychology, education, early years and nature and outdoor providers involved in the design process for the CONIFAS intervention suggested that co-design workshops should be child-led, with a focus on fun, whilst being meaningful and encouraging empowerment [10]. Child workshops for the design of that intervention were held at a nature reserve with a range of activity types to appeal to a variety of different children’s interests [10].

Usability testing was another method undertaken to gather children’s views. Hill et al. [24] who used this approach stressed that this ensured the mobile game application for children designed to be mediated by parents met the needs of children and was also easy to use and fun to participate [24].

Focus groups with children in primary schools along with consultations with children, parents and teachers involving drop-ins to local community groups and presentations of the intervention was a further approach used, though reflections on the acceptability were not noted [23].

Inclusivity was a key factor considered in choosing which children should be involved in the design process. Reflecting on the design process in an earlier pre-print version of their article, Cullingham et al. argued that “*involvement of people with lived experience in all stages of intervention development has allowed the integration of multiple and sometimes contrasting perspectives”* [62] ensuring that the “*approach ensured language used and the intervention itself were in-keeping with a neurodiversity-affirmative approach”* [62].

In Pile et al. study inclusivity and a broader range of inputs was enabled by providing virtual workshops were individuals found attending in-person difficult [10]. Alternative approaches to developing outputs were also considered with a graphic designer in this study producing visual summaries of the key points discussed so that they were more accessible to children [10].

Involving children in the design and evaluation of an intervention was reported by some study authors to be more time-consuming than a traditional researcher-led project [24, 25, 62]. However, researchers felt the value added through this approach more than compensated for the additional time taken and accommodating additional time for this during planning was recommended [25]. Studies reported active collaboration of children during the design stage was a *‘successful and important part of the development process’* [24] and *‘significantly contributed to the final design of the intervention and methodology’* improving feasibility and effectiveness of the intervention [23].

Sustaining engagement in co-design workshops over extended periods was a challenge though due to time pressures for families, even when they were adequately resourced (e.g. by providing food and refreshments) [10, 24]. Thus, attendance needs to be flexible and alternative methods, such as individual feedback sessions or alternative ways to input (e.g. via questionnaires, online sessions) need to be considered.

### Findings from child consultations

All the children involved in the participatory focus groups were able to talk about mental health (discussed generally as anxieties and worries), how this might feel, and they gave examples and made suggestions about what can help, demonstrating the capacities of children in this age group to discuss complex and sensitive concepts. Some of the children felt that the types of activities used in these interventions, for example the thoughts-actions-feelings triangle, could help children to calm down. They all agreed that they would be happy to do these activities with their parents, but some mentioned that it would be difficult if the things they were concerned or anxious about involved a parent. Some children then went on to make suggestions about who else they might talk to if there was a problem, such as a sibling or grandparent.

They also felt it was important for children to be involved in a private space without distractions to give their views and opinions. This space varied from child to child, with some preferring to be in their own space to talk. Others also felt that being active and able to play football or go for a walk as part of their activity would be important. Three boys explained how they use football to ‘calm down.’ Children felt that these types of intervention might give them more one to one time with a parent. They also suggested that it would help a parent to understand their child better by hearing how they feel.

The children typically preferred being in a group to give their opinion with people they knew – “*with a few people but not the whole class”*. They felt it was important to have time to get to know researchers first and build trust with them if they were going to help to develop an intervention. However, the relatively short time they spent with experienced participatory researchers conducting this review demonstrated how rapport can be effectively built with thorough preparation, a comfortable physical space that children feel welcome in, and access to a range of creative activities that they could partake in freely. Two children suggested that they would like to have a familiar toy to hold and cuddle when they were sharing their views and opinions. They all enjoyed having opportunities to write about their feelings and views. They felt it was important for children to be given a variety of ways to express themselves when giving their opinion, for example writing a letter or a note for a parent to read later. They particularly liked being able to draw, colour and use modelling materials. One participant suggested the use of emoticons (e.g., smiley or sad face) to show how they feel or whether they liked something. In their discussions, the children also emphasised that preferences vary from person to person, with some preferring to draw, some enjoy glueing and sticking, and others prefer creative writing or simply talking.

### Recommendations on how to involve children in intervention development and evaluation

Recommendations from this scoping review and child consultations include involving children early in the intervention design stage and making use of iterative co-design workshops that deploy creative and participatory activities that engage children in a meaningful way and help them express their opinions and views in ways that suit their needs and preferences. Examples include providing visual summarises and use of creative art-based activities to express opinions. Researchers should plan in opportunities to build rapport and trust with children as well as conduct co-design activities in familiar spaces, so children feel safe to express their views. Researchers should consider conducting co-design workshops for children separately to adults to ensure that their voice is heard. Involving children as evaluation respondents in feasibility trials and user testing can help to ensure that the parent-mediated intervention tasks are appropriate and acceptable to children. Where possible, children should be involved in development and evaluation activities who have lived experience of common mental health disorders and where possible experience of parent-led interventions to ensure meaningful engagement and participation in design. Researchers developing parent-led intervention and parent-mediated tasks should make use of creative and art-based, game-like and active ways to engage children in parent-mediated tasks, include pictures and visuals in manuals and books and offer opportunities for adaptability and personalisation where possible, including rewards for completion and progress made.

## Discussion

This review aimed to scope and map existing studies that have involved children in the development and evaluation of parent-led interventions that target common child mental health problems. Only four studies were found that involved children in the development of these types of intervention, with only one study involving participation of children under 8 years old [10]. Most of the included studies involved children in the evaluation of interventions and involved children only in reporting of changes in their own mental health outcomes. Common methods employed by studies which did involve children in the development of parent-led interventions included interviews, co-design workshops and feasibility testing of the interventions and their resources, but these had limited detail about learnings or outcomes of this involvement.

While findings in this area are limited, they indicate that involving children in the development and evaluation of parent-led interventions is possible. Benefits of this involvement include the development and refinement of interventions that so the parent-mediated tasks appeal to children with the potential to improve engagement and effectiveness. Iterative involvement may be most effective, rather than one-off consultations. Innovative participatory activities such as using creative and art-based methods to enable children to provide their opinions are preferred by children.

However, involving children in any stage of design and development of an intervention involves careful planning and longer lead times are needed to enable engagement (i.e. building rapport, relation building etc.) and methods for sustaining engagement need to be considered. While it is still unusual for younger children to be involved in development of interventions, findings of the one study that did involve children younger than 8 years old demonstrates that this can be done effectively [10], but consideration of providing accessible and engaging materials and activities is needed. Thus, age should not be used to limit involvement as young children demonstrate competency to talk about emotional states and wellbeing and express views on what sort of activities work [13, 14].

While there was little reported across the included studies about the outcomes of children’s involvement in design and development of parent-led interventions, children expressed preference for parent-mediated tasks within these interventions to be creative, involving art-based activities where possible, and game-like tasks. Children preferred parent-mediated tasks that enabled adaptability and personalisation and liked reward achievement for completion and progress. These insights, if acted upon, may well improve children’s engagement with parent-mediated tasks, influencing parents’ continuation with the intervention and therefore potential effectiveness for intended recipients.

There is limited evidence of what works in involving children in development of mental health interventions, but the findings of our review mirror those found in a review of digital interventions for child mental health [63]. Similar to the findings in our review, participatory co-design workshops and user-centred design methodology were found to be most effective. Children also similarly expressed a preference for personalisation within the intervention and liked rewards for completion of intervention components, indicating these are core elements for child-centred design. While that review found some intervention-specific (suitability, usability, and acceptability) and person-specific (motivation, capability, and opportunity) barriers to engagement with digital interventions they argue that involving children in the development of interventions helps to overcome these barriers by promoting usage, adherence, and positive user experience [63].

### Strengths and limitations

The rigorous methodological approach, in accordance with JBI Methodology for Scoping Reviews [30] and Preferred Reporting Items for Systematic Reviews and Meta-Analyses extension for Scoping Reviews (PRISMA-ScR) [31], was a key strength. In addition, the consultations conducted with children, enabled sharing the key findings from the research, allowing them the opportunity to reflect and present recommendations which added value to the findings from academic literature. However, this study highlights the paucity of involvement of children in the design and evaluation of parent-led mental health interventions and the lack of a strong evidence base of what works in this specific area.

Of those studies which did involve children, limited details were typically provided about methods and outcomes of involvement. Future research involving children should make use of reporting guidelines for patient and public engagement (i.e. GRIPP [64, 65]), to ensure that full details are given about how children were involved as well as reporting key findings from their involvement (including learnings about methods used). While tools (such as GRIPP) are useful to ensure researchers provide descriptive information about the participation and/or demographics of children involved, detailed articles where the focus is about learnings and reflection from participation methodologies itself will also be a valuable contribution to the field and help to build understanding of best practice for involving children in the development and evaluation of parent-led interventions.

A further limitation is that all included studies were conducted in high-income, developed countries. As a result, the findings may not fully reflect the experiences, structural conditions, or cultural contexts present in low- and middle-income countries, which may limit the global applicability and transferability. While the review was not limited by country only studies in developing countries were identified, this may be due to the English language restriction or that there have been no studies conducted that involved children in development and evaluation of parent-led mental health interventions in these countries. A recent systematic review of child and adolescent mental health outcomes of parenting and family interventions in low to middle income countries which did not apply any language restrictions found effectiveness of interventions targeting parent, carer and families to support children’s mental health in these countries [66]. While this review focussed on children’s involvement in these interventions rather than their effectiveness, it will be important in future work to include publications in languages other than English to enable any available learnings from these countries to be included and enable best practice suggestions for involving children to incorporate cross cultural considerations and contexts.

There was also a lack of ethnic and cultural diversity in the included studies. Just under half of the studies did not report ethnicity and where it was reported most studies lacked diversity. Cultural factors were largely unreported. As a result, the findings in this review may not be generalisable and important considerations relating to inclusivity may be unrepresented.

A further limitation is that we did not include dissertations. While we did not find any relevant studies in grey literature reports it is possible that there may have been relevant research that has not been published in academic journals that was missed by not including other grey literature.

Overall, the review found that children’s involvement in the development and evaluation of parent-led interventions for child mental health problems is very limited. This reflects a broader systemic tendency to prioritise parental perspectives without fully considering children’s lived experiences or priorities. For example, Chapman et al. [67] conducted a systematic review of randomised controlled trials of psychological interventions targeting only parental anxiety [67]. They found no trials in which child mental health or wellbeing outcomes were assessed.

Co-design offers a way to counter this tendency by ensuring that intervention goals, content, and outcome measures reflect what matters to children: how they feel at home, how their relationships with parents shift, what kinds of support they value, and what changes they hope to see. Children can also provide insight into practical aspects of parent-mediated intervention strategies (such as how parents support children’s coping skills, set routines, manage behaviour, or scaffold problem-solving), which can make these more engaging, comprehensible, and relevant to children and their everyday family lives. Children’s involvement is not simply a participatory ideal; it is a practical and essential means of improving intervention quality, real-world fit, and the likelihood of meaningful impact on child mental health.

The findings of this review highlight several important implications for clinical practice and intervention development. First, children’s perspectives offer unique insights into what makes parent-led interventions engaging, acceptable, and meaningful, suggesting that routine involvement of children in early design stages could strengthen the relevance and feasibility of these approaches. The emphasis children placed on creative, interactive, and sensory activities indicates that clinicians and developers should integrate child-friendly methods that support emotional expression and sustained engagement. In addition, relying solely on parent-report may overlook children’s internal experiences; incorporating child-centred outcome measures and structured opportunities for children to provide feedback could enhance the accuracy and usefulness of clinical evaluations.

## Conclusions

Very few studies involve children in the development and evaluation of parent-led interventions other than capturing self-report data. Common methods from emerging studies where children are involved included interviews to understand more about mental health difficulties from a child’s perspective, co-design workshops to discuss intervention components, and feasibility testing of intervention resources. Recommendations from review findings and participatory focus groups with children included early involvement in the design, more interactive, inclusive, and innovative child-specific co-design workshops, and involving children with lived experience when designing or evaluating a health-specific intervention. Finally, if participatory approaches within research do become more integrated in typical practice, future research should include greater detail on the methods and effectiveness, to enable a more extensive understanding of best practice in how to involve children in the design, development and evaluation mental health interventions.

## Supplementary Information

Below is the link to the electronic supplementary material.

Supplementary Material 1 (DOCX 108 KB)

Supplementary Material 2 (DOCX 283 KB)

Supplementary Material 3 (DOCX 14.3 KB)

Supplementary Material 4 (DOCX 167 KB)

Supplementary Material 5 (XLSX 80.0 KB)

## Data Availability

The dataset supporting the conclusions of this article is included as an online resource (online resource 5).
