# Supplementary material for: Children’s participation in the development and evaluation of parent-led interventions for children’s mental health: a scoping review and consultation with children
Source: Eur Child Adolesc Psychiatry. 2026 Sep 15;35(11):3409–25. doi: 10.1007/s00787-026-03187-0 (PMC13710420; doi:10.1007/s00787-026-03187-0)
Supplement: Supplementary file 2 [file 787_2026_3187_MOESM2_ESM.docx]

Children’s participation in the development and evaluation of parent-led interventions for children’s mental health: A scoping review and consultation with children, European Child & Adolescent Psychiatry, Nowland et al., Corresponding author: Rebecca Nowland, [rebecca.nowland@manchester.ac.uk](mailto:rebecca.nowland@manchester.ac.uk)

**Online Resource 1 - Search strategy (Medline – OVID)**
