# Supplementary material for: Children’s participation in the development and evaluation of parent-led interventions for children’s mental health: a scoping review and consultation with children
Source: Eur Child Adolesc Psychiatry. 2026 Sep 15;35(11):3409–25. doi: 10.1007/s00787-026-03187-0 (PMC13710420; doi:10.1007/s00787-026-03187-0)
Supplement: Supplementary file 3 [file 787_2026_3187_MOESM3_ESM.docx]

Children’s participation in the development and evaluation of parent-led interventions for children’s mental health: A scoping review and consultation with children, European Child & Adolescent Psychiatry, Nowland et al., Corresponding author: Rebecca Nowland, [rebecca.nowland@manchester.ac.uk](mailto:rebecca.nowland@manchester.ac.uk)

**Additional file 2 – Key Organisation Website Searching**

Mental Health Foundation

Youth endowment Fund

Children’s Society

Youth Action

Mind

McPin

Young Minds

Involve

Charlie Waller

Anna Freud Centre

Cambridge Curiosity and Imagination

NSPCC

National Autistic Society

Autistica

Joseph Rowntree Foundation

Health Foundation

Place2Be

Barnardo's
