# Supplementary material for: Children’s participation in the development and evaluation of parent-led interventions for children’s mental health: a scoping review and consultation with children
Source: Eur Child Adolesc Psychiatry. 2026 Sep 15;35(11):3409–25. doi: 10.1007/s00787-026-03187-0 (PMC13710420; doi:10.1007/s00787-026-03187-0)
Supplement: Supplementary file 4 [file 787_2026_3187_MOESM4_ESM.docx]

Children’s participation in the development and evaluation of parent-led interventions for children’s mental health: A scoping review and consultation with children, European Child & Adolescent Psychiatry, Nowland et al., Corresponding author: Rebecca Nowland, [rebecca.nowland@manchester.ac.uk](mailto:rebecca.nowland@manchester.ac.uk)

**Online Resource 3 – Schedule for Consultations with Children**

Children’s voice in parent-led mental health interventions

Plan for focus group with children

3 groups of children (5-6 in each group) – approximately 1 hour each session, depending on school timetable

**Meeting with school prior to session** – The plan will be shared and agreed with school prior to the session. Check safeguarding and support available and referral etc., ask about basic protocols and key staff (i.e. do children know who the school counsellor is, how might they refer to this person).

Flexible session – children can respond verbally, written or through drawing. Researchers to build rapport and encourage engagement and input flexibly according to their needs etc. Researchers to bring a fluffy animal to pass round to help children with taking turns to speak/contribute.

Resources – small world figures, modelling equipment, paper, pens, paint etc.

Plan for session

1. **Introductions** – who we are? Explain we are researchers and want to find out about mental health of children and young people and how to help them. Discuss consent as per the Child consent checklist (using appropriate language).
2. **Ice breaker activity** – first letter of name and favourite animal, animal with the same first letter or animal that represents you – actions to represent the animal (dependent children’s level of engagement/confidence).
3. **Explain about worries and anxieties and helping strategies** - We are going to talk about how children get help when they are feeling anxious or worried. What do you do when you are getting a bit worried? How do your parents do to help you when you have worries? Who else might help you? (Q and A session)
4. **Explain about the therapy** – To help children with mental health (prompt if needed - anxious, worried, upset) sometimes you might go to see a doctor or another person that is an expert in children’s mental health and they will help you to cope with your worries and anxieties.

But also sometimes these people (doctors or other professionals) help children by teaching your parents how to help you to cope with these anxieties and worries. Then your parents help you by doing activities at home. Shall we have a go at looking at some of these activities:

5. **Sharing activities from the interventions** - Children to be shown and talked through the activities as a group rather than to complete them and researchers to facilitate a discussion with children using the questions below.

Body map

CBT triangle

What is the first thing you thought when we showed you these activities?

The children that have been doing these activities haven’t been asked about what they think or feel about doing these with their parents. We think it is really important that children have a say in this, so we want to hear how you think this should happen. What did you think of these activities? How would you feel about your parents helping you with these activities? What do you think would be the best way to let people know if you didn’t like an activity you were asked to do?

What do you think of doing these activities on paper like this? How else could they be done? What would you like?

Do you think it is fair that parents learn all this and then help you? Is it okay that you learn by your parents showing you or would you like to learn from the doctor or health person? Should parents and children go together? Why?

**Ways to find out from children what they think**

We want to ask lots of other children what they think about these types of activities. How do you think we should do that? Prompt: when people have done this before they have – used focus groups (like we are doing today with you), interviews (talking to children about their experiences on their own rather than in a group) and children have tried out the activities on the computer.

Children said these things when they were interviewed (show children quotes on cards from previous studies):

An 11-year-old child stated, “*It was kind of good because I got to be with my mommy and talk to her about things that were on my mind*.”

A 12-year-old stated, “*It was actually pretty amazing to actually write about my feelings, and then I decided to make my own notebook to write my own thoughts*.”

A 12-year-old reported, “*Um, we’ve gotten more closer from talking*.”

How do you like to give your opinion? When we ask children for their opinion we sometime do creative things – like ask children to write a story or draw a picture – how do you like to let someone know what you think?

**Planning for an evaluation** - Group activity

Flipchart paper – let’s imagine we want to plan for a timetable of things to do to get children’s views about these activities – what should be planned to do with children? How should we do it? What would be important for the adults to do so that you are comfortable? (draw/write this out together).
